# Characterization of the bovine pregnancy-associated glycoprotein gene family – analysis of gene sequences, regulatory regions within the promoter and expression of selected genes

**DOI:** 10.1186/1471-2164-10-185

**Published:** 2009-04-24

**Authors:** Bhanu Prakash VL Telugu, Angela M Walker, Jonathan A Green

**Affiliations:** 1 Division of Animal Sciences, University of Missouri, Columbia, MO 65211, USA; 2 Department of Veterinary Pathobiology, University of Missouri, Columbia, MO 65211, USA; 3 Christopher S. Bond Life Sciences Center, University of Missouri, Columbia, MO 65211, USA

## Abstract

**Background:**

The Pregnancy-associated glycoproteins (PAGs) belong to a large family of aspartic peptidases expressed exclusively in the placenta of species in the *Artiodactyla *order. In cattle, the *PAG *gene family is comprised of at least 22 transcribed genes, as well as some variants. Phylogenetic analyses have shown that the PAG family segregates into 'ancient' and 'modern' groupings. Along with sequence differences between family members, there are clear distinctions in their spatio-temporal distribution and in their relative level of expression. In this report, 1) we performed an *in silico *analysis of the bovine genome to further characterize the *PAG *gene family, 2) we scrutinized proximal promoter sequences of the *PAG *genes to evaluate the evolution pressures operating on them and to identify putative regulatory regions, 3) we determined relative transcript abundance of selected *PAGs *during pregnancy and, 4) we performed preliminary characterization of the putative regulatory elements for one of the candidate PAGs, *bovine *(*bo*) *PAG-2*.

**Results:**

From our analysis of the bovine genome, we identified 18 distinct *PAG *genes and 14 pseudogenes. We observed that the first 500 base pairs upstream of the translational start site contained multiple regions that are conserved among all *boPAGs*. However, a preponderance of conserved regions, that harbor recognition sites for putative transcriptional factors (TFs), were found to be unique to the modern *boPAG *grouping, but not the ancient *boPAGs*. We gathered evidence by means of Q-PCR and screening of EST databases to show that *boPAG-2 *is the most abundant of all *boPAG *transcripts. Finally, we provided preliminary evidence for the role of ETS- and DDVL-related TFs in the regulation of the *boPAG-2 *gene.

**Conclusion:**

*PAGs *represent a relatively large gene family in the bovine genome. The proximal promoter regions of these genes display differences in putative TF binding sites, likely contributing to observed differences in spatial and temporal expression. We also discovered that *boPAG-2 *is the most abundant of all boPAG transcripts and provided evidence for the role of ETS and DDVL TFs in its regulation. These experiments mark the crucial first step in discerning the complex transcriptional regulation operating within the *boPAG *gene family.

## Background

Duplication of a chromosomal region containing a gene results in two copies of the parent gene. In most cases, purifying selection on both members of the gene pair remains until one of the paired genes acquires an inactivating mutation(s) and undergoes degeneration to a pseudogene. Typically, the pseudogene will eventually disappear from the genome due to chromosomal remodeling and/or locus deletion. This process is known as 'non-functionalization' [1]. While the genomes of all multicellular eukaryotes have evidence for such pseudogenes, there are also cases where alterations have occurred within coding or regulatory sequences allowing the formerly redundant gene to take on entirely new functions. This process is known as 'neo-functionalization'[2]. Neo-functionalization of a duplicated gene is rare. A distinct and more likely alternative is 'sub-functionalization' [3,4]. According to this model, complementary partial 'loss-of-function' mutations in both genes results in the sharing of a full complement of functions that had been performed originally by the single ancestral gene [2]. It has been emphasized that the changes that lead to sub-functionalization probably happen primarily at the level of regulatory regions of the promoter, rather than happening in the coding region to directly influence protein function [5]. Alterations in regulatory elements could produce discrete expression patterns that, together, would recapitulate the more complex expression pattern of the ancestral gene [6]. Therefore, the end result of neo- and sub-functionalization is the birth of novel gene pairs that can subsequently be expanded into larger gene families [2,5].

All sequenced mammalian genomes to date have revealed, among other things, a predominance of multi-gene families whose products are expressed in reproductive organs such as the placenta [7-10]. Within the placenta, the chorionic trophoblasts constitute the outer fetal-derived cells that form the interface between the maternal uterus and the fetus. They perform a range of transport and endocrinological functions that provide support to the growing fetus. At times, the physiological demands of the fetus are not necessarily compatible with the interests of the mother to provide for current and future offspring [11]. Consequently, the potential exists for genetic conflict between these individuals. As part of the interface between the fetus and the mother, gene products that are functional in trophoblast are targets of selective pressures arising from this so called 'genetic arms race'. These selective pressures are thought to drive the birth of novel gene families [8]. Indeed, examples of such gene families do exist in the placenta of domestic cattle and other ruminant ungulates. One example is a family known as the *pregnancy-associated glycoproteins *or *PAGs*.

*PAGs *represent one of the major trophoblast secretory products of species in the *Artiodactyla *order (swine, cattle, deer, camels, etc) [12-17]. The PAGs are related to the aspartic peptidases (APs), a diverse grouping that includes gastric enzymes such as pepsin and chymosin, lysosomal enzymes such as cathepsin D, and the enzyme renin, which is critical for maintaining salt homeostasis and blood pressure [18]. Mammalian APs are strikingly conserved in regard to their gene structure (most mammalian aspartic peptidase genes consists of nine exons and eight introns) [18-23]. The APs also have a conserved bi-lobed structure [18,24-26] with the two lobes of the enzyme being roughly symmetrical and enclosing a substrate binding cleft between them. Positioned within this cleft is a catalytic center that contains two aspartic acid residues (one from each lobe) flanked by conserved amino acids [18,27]. These aspartates participate in the catalytic mechanism.

In species within the *Ruminantia *suborder, the *PAGs *constitute a large and diverse family [12-16,28]. In cattle, for example, 22 distinct *PAG *cDNAs have been deposited in Genbank, in addition to some closely related variants. However, the exact number of *PAG *genes remains a mystery. The *PAGs *that have been cloned in cattle fall into two main groupings: an evolutionarily more 'ancient PAG' group, whose members are transcribed in all cotyledonary trophoblast cell types, and a second group that arose more recently (the 'modern PAGs'). These modern PAGs are transcribed exclusively by a specialized subset of trophoblasts called binucleate cells (BNC) [13,15,29,30]. Interestingly, many of the *PAGs *in the modern-grouping have amino acid substitutions at conserved positions within the catalytic center that may preclude these *PAGs *from functioning as enzymes [14,27,31-33]. The ancient PAGs on the contrary, possess all the hallmarks of typical aspartic peptidases and are predicted to be active enzymes [27]. It is also worth noting that the *PAGs *are not expressed coordinately throughout pregnancy. Some are expressed early, while others are not detectable until later in the pregnancy [13,34].

The sequencing and ensuing assembly of the bovine genome has provided two unique opportunities. One has been the opportunity to identify and evaluate all the potential *PAG *and *PAG-like *genes represented in the genome build. The other advantage was to gain access to full length sequences of the *PAG *genes, especially their promoter and regulatory regions, thus facilitating analyses and insight into *PAG *transcriptional regulation. The goal of this current work was to take advantage of both these opportunities to perform an *in silico *analysis to annotate the *PAG *genes within the bovine genome, as well as their promoter regions. Particular attention was focused on the regulatory elements of *bovine (bo) PAG-2*, which appears to be the most abundant *PAG *transcript, and to characterize its relative expression compared to other ancient *PAGs*.

## Methods

### Annotation of *PAGs *within the bovine genome (Build 3.1)

To annotate the *PAG *genes in the bovine genome, guidelines set forth for annotation by the bovine genome consortium were followed. An official gene set for the bovine genome, termed the GLEAN set, was developed by the consortium using a powerful gene prediction algorithm [35], and was provided to the manual annotation community. The first step in the manual annotation of *boPAGs *was to identify the closest GLEAN model for each candidate gene listed in Table 1 that were available through RefSeq or Genbank (if RefSeq was not available). The nucleotide sequence of each known *boPAG *was used as a query in BLAST searches in the bovine genome consortium web browser. The GLEAN sequence was then visually inspected through the Apollo Genome Annotation and Curation Tool (v.1.6.5) [36], for the presence of a putative translation start site, intact exon-intron boundaries and defined 5' and 3' UTRs [36,37]. More importantly, the open reading frame (ORF) was scrutinized thoroughly for any mismatches with known RefSeq or cDNA clones by performing megaBLAST searches (an algorithm for highly related nucleotide sequences) in the NCBI bovine genome database and a BLASTN search in the bovine genome browser (GBROWSE) .   [38]. Any incompatibility was further evaluated by performing megaBLAST against the bovine EST database in Genbank. The sequence with the best EST support was accepted. Based on these analyses, GLEAN models were accepted, rejected, or refined. The final models were submitted to the genome annotation curators for independent review by the consortium.

**Table 1:** Summary of results from the *in silico *analysis of *PAG *genes in the bovine genome.

**Gene name**	**PAG grouping**	**Transcript ID**	**REFSEQ number/** **Temporary Gene ID**	**GLEAN**	**Chromosome** **location**	**Comment**	**Missing exons**
*BOPAG-1*	Modern	M73962	NM_174411	**10324**	ChrUn.13	full-length	
*BOPAG-2*	Ancient	L06151	NM_176614	**19448**	Chr29.48	full-length	
*BOPAG-2 like*	Ancient	XM_001252523	Temporary ID:786	**10441**		predicted; partial	Partial exon-6
*BOPAG-3*	Modern	XM_615231	Temporary ID:2418	**24765**	Chr29.54	full-length	
*BOPAG-4*	Modern	AF020506	NM_176615	**10334**	ChrUn.13	full-length	
*BOPAG-4 like*	Modern	XM_600174		**24769**		predicted; full length	
*BOPAG-5*	Modern		NM_176616	**18735**	ChrUn.278	full-length	
*BOPAG-5 like*	Modern	XM_001252636	Temporary ID:3317	13899	ChrUn.1071	predicted; full length	
*BOPAG-5 like*	Modern	XM_001252636		**10439**	ChrUn.833	predicted; full length	
*BOPAG-5 like*	Modern	XM_598365		10317		predicted: partial	Missing Exons 1–2
*BOPAG-6*	Modern	AF020508	NM_176617	**24763**	Chr29.54	full-length	
*BOPAG-7*	Modern	**BC133469**		NR		full-length	
*BOPAG-7 like*	Modern		**NM_176618**	NR		Splice variant	
*BOPAG-7-like*	Modern		NM_001109978	13897	ChrUn.1071	predicted; partial	Missing Exon 6
*BOPAG-8*	Ancient	AF020510	NM_176619	**24771**	Chr29.54	full-length	
*BOPAG-9*	Modern	AF020511	NM_176620	**18917**	ChrUn.1099	full-length	
*BOPAG-10*	Ancient	AF020512	NM_176621	**19477**	Chr29.48	full-length	
*BOPAG-10-like*	Ancient	XM_864803	Temporary ID:2428	19475	Chr29.48	predicted; partial	Misisng Exon 9
*BOPAG-10-like*	Ancient	XM_864803	Temporary ID:2805	19476	Chr29.48	predicted; partial	Misisng Exon 9
*BOPAG-11*	Ancient	AF_020513	**NM_176623**	24761;24762	Chr29.54	full-length	
*BOPAG-12*	Ancient	AF_020514	NM_176622	**19478**	Chr29.48	full-length	
*BOPAG-12-like*	Ancient		Temporary ID:3319	10442	ChrUn.833	predicted;partial	Partial exon 5
*BOPAG-13*	Ancient	AF_192330		NR		not represented	
*BOPAG-14*	Modern	**AF_192331**		NR		not represented	
*BOPAG-15*	Modern	AF_192332	NM_176624	**10338**	ChrUn.13	full-length	
*BOPAG-16*	Modern	AF_192333	NM_176625	**10332**	ChrUn.13	full-length	
*BOPAG-16-like*	Modern	XM_596391	Temporary ID:391	10319	ChrUn.13	predicted;full length	
*BOPAG-17*	Modern	**AF_192334**	NM_176627	17225*		* Model error	
*BOPAG-17-like*	Modern	XM_001252975	Temporary ID:2324	**10321**	ChrUn.13	predicted;partial	Missing exon 5
*BOPAG-18*	Modern	AF_192335	NM_176626	**18733**	ChrUn.278	full-length	
*BOPAG-19*	Modern	AF_192336	NM_176628	**10322**	ChrUn.13	full-length	
*BOPAG-19-like*	Modern	XM_001253033	Temporary ID:535	**10323**	ChrUn.13	predicted;full length	
*BOPAG-19-like*	Modern		Temporary ID: 2015	10327	ChrUn.13	predicted:partial	Misisng Exon 9
*BOPAG-19-like*	Modern		Temporary ID:994	10328	ChrUn.13	predicted;partial	
*BOPAG-20*	Modern	AF_192337	NM_176629	**10330**	ChrUn.13	full-length	
*BOPAG-21*	Modern	AF_192338	NM_176630	**10329**	ChrUn.13	full-length	
*BOPAG-22*	Modern	AY911498		NR		not represented	

* Model error: The GLEAN model has an error. The displayed sequence does not match the GLEAN model sequence.

NR: GLEAN model was not represented in the bovine genome build.

In addition to the known *boPAG *genes, other putative *PAG-like *genes were present among the GLEAN models. These predicted genes were queried with cross-species megaBLAST and BLASTN against the bovine EST database and the nucleotide collection (nr/nt) database in Genbank to help determine if these predicted genes are actively transcribed.

### Phylogenetic relationships of *boPAG*-genes

The translated sequences for the *boPAG *genes used to establish phylogenetic relationships within the *PAG *gene family were displayed in bold in Table 1. All annotated *boPAGs *and the *boPAG *genes with known mRNA sequence (but not represented in bovine genome assembly, 3.1) were included in the analyses. The variants of *PAG *genes with a conserved nine exon structure, splice variants, and those transcripts with one missing exon were also included in the analysis.

The translated sequences were aligned by pairwise comparisons by using CLUSTALW in BioEdit version 7.09 [39,40]. Phylogenetic analysis of the aligned sequences were performed by using the MEGA4 program [41]. All positions within the alignment that contained gaps or missing data were ignored during pairwise comparisons. An initial tree was generated by using the Neighbor-Joining method [42], followed by Minimum Evolution [43] and bootstrapping tests (n = 1000 replicates). The inferred consensus tree was displayed [44].

### Analysis of repeat elements within *boPAGs*

RepeatMasker, version 3.1.9 [45] was used to scan for inserted transposable elements (TE) in the entire gene sequence as well as 3000 bp upstream of the translational start site (TSS-ATG), and 3000 bp downstream of the translational termination codon (TAA, TGA and TAG) of each representative *boPAG *gene. The parameters used for the analysis were described elsewhere [46]. Briefly, 'cross match' was used as the search engine, cow (*Bos taurus*) was identified as the DNA source, simple repeats and low complex repeats were requested not to be masked and the matrix was set to sub-loci optimization pre-runs.

### Analysis of proximal promoter sequences

#### Investigation of selective pressures operating on the proximal promoter sequence of *boPAGs*

The selective pressures operating on the ORFs of various *boPAG *genes have been analyzed systematically in prior publications [29,32]. The availability of full length gene sequences has made it possible to extend similar types of analyses to the *PAG *promoter regions. Two different lengths of promoter sequence were chosen for comparison [1000 bp as well as 500 bp proximal to the TSS] between several ancient *boPAGs *(*boPAG-2, 8, 10, 11 *and *12*) and some representative modern *boPAGs *(*boPAG-1, 3, 4, 5, 6, 7, 15, 18, 19, 20 *and *21*) to simplify the analysis. The nucleotide sequences were aligned by using CLUSTALW in the MEGA4 software suite. All the deletions and gaps arising from the alignment were eliminated by using the pairwise deletion option. The aligned *boPAG *sequences were subjected to pairwise comparisons in MEGA4 by using the Maximum Composite Likelihood method with 1000 bootstrap replicates to calculate the p-distance (number of differences/total length of sequence analyzed).

In order to understand the type of evolutionary pressures operating on the promoter regions, we plotted the inferred p-distances obtained from the promoter analysis against the proportion of synonymous changes per synonymous site (dS) estimated for the corresponding *boPAG *ORFs. The underlying assumption for this approach was that, dS within the ORFs would approximately reflect the rate of nucleotide change in the locus in the absence of selection. In other words, if the p-distance of the promoter equals dS of the corresponding exons of the gene (p-distance/dS = 1), then the *boPAG *promoter is accumulating substitutions in this region at a rate that corresponds to that expected, based on normal mutation rates. A value >1 would indicate that nucleotide changes are occurring faster than would be predicted and a value <1 would suggest stringent purifying selection, with fewer substitutions being tolerated and hence retained.

### Multiple sequence alignment of the proximal promoter regions of selected *boPAG *genes for identification of incorporated Transposable elements (TE) and conserved regulatory regions

#### Identification of TE in the proximal promoter region

To explain for the apparent disparities in evolutionary pressures operating on the non-coding proximal promoter sequences of the *boPAG *genes, 1000 bp upstream of the translational start codon (ATG) were aligned with CLUSTALW. Within this alignment, insertions of TE, identified by the repeat masker program, were visually detected and mapped to the *boPAG *promoter sequences.

#### Identification of putative transcription factor (TF) binding sites in the proximal promoter region

DiAlign TF, a component of the comprehensive promoter analysis software, Genomatix GEMS launcher [47], was used to align and search for putative transcription factor (TF) binding sites within the proximal promoter regions of select *PAGs*. Approximately 1000 bp upstream of the TSS (proximal promoter) of eight *boPAGs *[4 ancient (*boPAG-2, -8, -11*, and -*12*), and 4 modern (*boPAG-3, -5, -15*, and -*18*)] that were recognized by the GEMS database were used in the analysis. The following parameters were selected for performing the analysis: Matrix library 7.0 was used as the default library to match the TF binding sites, and 'all' the matrix groups from 'embryo' tissue type were selected as a reference. Input sequences were aligned and regions closely matching known TF-binding sites that were conserved in more than 50% of the input sequences (4 out of 8) were mapped. The output from the analysis was modified and presented in multiple sequence alignment with artificial shading to facilitate easier comprehension.

#### Estimation of relative frequency of various *boPAG *ESTs found within the bovine genome

In order to estimate, how differences within the *boPAG *promoters reflect *in vivo *expression differences, relative levels of transcription were determined based on the representation of each gene in common bovine EST databases. Known *boPAG *cDNAs were each queried by BLASTN in the NCBI bovine EST database. ESTs that exceeded 98% in identity in at least 350 bp of query nucleotide sequence were considered to be a positive match with a particular *PAG*.

#### Quantitative Real-time PCR of ancient boPAGs (*boPAG -2, -8, -10, -11 *and -*12*)

It was noted from the analysis of the proximal promoters and EST frequencies, that there were some distinct differences in both the TF-binding sites within the regulatory regions and the EST frequencies of the *boPAGs*, particularly among the ancient *boPAG *members. Such differences in putative regulatory elements were even observed between two closely related ancient *boPAG *members (*boPAG-2 *and -*12*). In order to determine if these minor differences in the purported promoter elements can influence the relative expression of the *boPAGs*, quantitative Real-time PCR (Q-PCR) was performed to monitor relative transcript abundance of the ancient *PAGs *in placental RNA harvested from different stages of pregnancy.

RNA was extracted from placental cotyledons at various stages of pregnancy (days 45, 60, 75, 90, 140, 170, 220 and 280) by using STAT-60 RNA extraction reagent (IsoTex diagnostics, TX, USA). Each gestational stage was represented by two different animals. The extracted RNA preparations were treated with amplification grade DNAse I (Invitrogen, CA, USA) at room temperature according to the manufacturer's recommendations. The DNA-free RNA samples were quantified and analyzed for quality (260/280) and agarose gel electrophoresis. Two micrograms of high quality RNA from each sample were reverse transcribed by using an oligo-dT primer and SuperScript III-reverse transcriptase (Invitrogen, CA, USA) at 50°C for 1 hr.

Oligonucleotides for Q-PCR were designed to span exons of each *boPAG *to prevent unwarranted amplification of any trace carry-over contamination from the genomic DNA. Oligonucleotides were also designed for a control gene in cattle, *YWHAG *(*tyrosine 3-monooxygenase/tryptophan 5-monooxygenase activation protein, gamma polypeptide*). Power SYBR^® ^Green PCR master mix (Applied Biosystems, CA, USA) reagent and the Applied Biosystems ABI Prism 7500 Real-Time PCR system were employed for the Q-PCR. The reaction conditions for the Q-PCR were optimized by determining the amplification efficiency, as well as the dynamic range for each primer set, according to methods described by the manufacturer. Following the preliminary evaluation, the optimum oligonucleotide sets were selected (Table 2). The Q-PCR for each candidate gene was performed with two biological replicates and duplicate technical replicates. The cycling conditions were: pre-heating for: 50°C for 2 min (1 cycle); followed by a pre-run to activate the polymerase at 95°C for 10 min (1 cycle) followed by 40 cycles of 95°C for 15 sec, 65°C for 30 sec and 75°C for 1 min, with the data being acquired in the 75°C window. The data was analyzed by the ABI-PRISM 7500 sequence detection system software and the results from the analysis were graphed.

**Table 2:** The oligonucleotides used for quantitative PCR to measure relative transcript abundance of ancient bovine PAGs during pregnancy.

**PAG**	**Sequence**	**Primer location (bp)**	**Amplicon size (bp)**
*BoPAG-2*	F: 5'-GTAGGCTCGCCTATCACCATCTTC-3'	360	289
	R: 5'-CCTCTGGCTTGTTTGTGTTCAAGTAG-3'	649	
*BoPAG-8*	F: 5'-CCTATCCTGAATGACGAGCAA-3'	886	128
	R: 5'-CCTTTCAGAAAACCTCTGGATG-3'	1014	
*BoPAG-10*	F: 5'-TTGAGCAGTCAGAAAGAGAACG-3'	631	136
	R: 5'-TTCATGGAGATGCTGTCTATGTTT-3'	767	
*BoPAG-11*	F: 5'-CGGTTCCGAGTACATGGTTT-3'	885	114
	R: 5'-AGAAATCTTTTGGATGTAGGCTTC-3'	999	
*BoPAG-12*	F: 5'-ACACACCAGCCTATTAGCATCTCC-3'	361	266
	R: 5'-CGGCTGGCATGTGTTCAAGTAG-3'	627	
*YHWAG*	F: 5'-AGCACATGCAGCCCACTC-3'	488	120
	R: 5'-TCGTCGAAGGCGGTCTTG-3'	608	

#### Electrophoretic mobility shift assays (EMSA) to evaluate the role of ETS-2 and conserved repeats in the *boPAG-2 *promoter

Since *boPAG-2 *was established as the most abundantly transcribed of all the known *PAGs*, EMSAs were performed to determine if some of the elements conserved in the regulatory regions were capable of binding to putative TFs. Oligonucleotides (IDT, IA, USA) were designed to encompass sequences in the regulatory region that were predicted to be involved in transcriptional regulation of the *boPAG-2 *gene. The sequence of the probe encompassing the putative ETS site is: CCTCAAGG*AAGA*GATCACAG. The predicted binding site for ETS is shown italicized in the oligonucleotide sequence. This site corresponds to base positions -227 to -230 in the aligned sequence. The oligonucleotides used to examine binding for the unique repeated regions in the promoter are: GTTAACAAGT*TTCTCCA*TGC (BR1) and TATT*TTCTCCA*AGTTAACAAG (BR2). These unique repeats, which are shown italicized in the sequence, correspond to -284 to -291 and -302 to -311, in the aligned sequence. The oligonucleotides were annealed and end-labeled with [^32 ^P-γ] ATP by using T4 poly nucleotide kinase. Binding reactions were performed by using radiolabeled probe (10,000–20,000 cpm/25 fmol) with 20 μg of JAr choriocarcinoma cell nuclear extracts in the presence of 1 μg of nonspecific competitor (poly dI:dC; Sigma, MO, USA). The nuclear extracts for EMSA were prepared as described by Dignam et al. [48]. The composition of the buffer used was 20% (v/v) glycerol, 5 mM MgCl2, 2.5 mM EDTA, 2.5 mM dTT, 250 mM NaCl, 50 mM Tris-HCl of pH 8.0 containing 2% (v/v) CHAPS detergent and 10 mg/mL BSA (Sigma, MO, USA). For competition assays, a 50–250 molar excess of unradiolabeled competitor DNA (cold probe) was used. The ETS- 2 antibody competition assays were performed by mixing 2 μg of ETS antibody (Santa Cruz Biotechnologies, CA, USA) with 20 μg of nuclear extracts. The mixture was incubated on ice for 30 min followed by addition of the radiolabeled probe and incubation at room temperature for 30 min.

## Results and discussion

### The *PAG *gene family in cattle

The *PAG *gene family in cattle was found to be relatively large. A total of 22 distinct *PAG *cDNAs have been deposited into GENBANK, in addition to numerous variants and pseudogenes, which underscore the complexity of this gene family in the bovine genome. Of the 22 *boPAG *cDNAs, one transcript *boPAG-22 *is a variant of *boPAG-2 *and is not distinct enough to be categorized as a separate *boPAG*. However, we included *boPAG-22 *in our initial analysis. Needless to say, the annotation of such an extensive gene family is prone to errors in the assembly because of the repetitive nature of duplicated genes which are often arranged in tandem. Therefore, one of the principal objectives of this report was to annotate the *PAG *genes within the currently available bovine genome build (3.1). The results were compiled and displayed in Table 1. The table contains the accession numbers for representative cDNA, the corresponding RefSeq transcript/Bovine Genome temporary Gene ID, in addition to the GLEAN model best matching the sequence. Locations of these transcripts on the chromosomal scaffolds are also indicated. In the table, "Chr Un." indicates that the gene is unassigned to any specific chromosomal scaffold. Out of the 37 potential *boPAG *genes (known and predicted), there were 18 full length functional *PAG *genes that were represented and properly annotated in the genome assembly (build 3.1). Four *boPAG *genes, *boPAGs*-*7, 13, 14 *and -*22*, previously described based on cDNA cloning, were not represented in the build. There were three *boPAG-like *genes that were predicted by the *in silico *gene prediction analysis as having the conserved 9-exon structure of *PAGs *(GLEAN-IDs: 24769, 10319 and 10323). One of the putative genes, the *boPAG-19 like *gene (GLEAN_10323) had 100% identity with the *boPAG-19 *gene, both in the ORF and the proximal promoter regions. Therefore, it is presumed that this gene is a recently duplicated copy of the *boPAG-19 *gene. The other two predicted genes were not shown to be actively transcribed. Along with the full length *boPAG*-*like *genes, there were an additional 12 predicted genes that seemed to be incomplete (e.g. missing exons) (Table 1). The *boPAG-like *genes that are missing exons are likely pseudogenes because no ESTs were found that matched these sequences (data not shown). We consider 18 intact genes to be a conservative estimate of the actual number of *boPAG *genes since some known *boPAGs *were not represented in the build and we could not rule out the possibility of additional *PAG-like genes *that may have been unrecognized and not included in the assembly. All the annotated *boPAG-genes *that were assigned to a chromosome location were found to be clustered on chromosome 29.

### Evolutionary relationships of *PAG *genes in cattle

**Figure 1:**
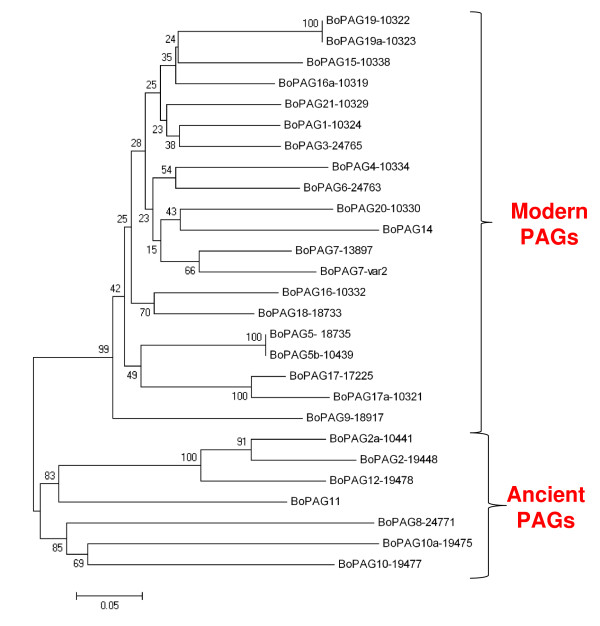
**Evolutionary relationships among the bovine *PAG *genes represented in the bovine genome build 3.1**. The tree was created from the inferred amino acid sequences by using the Minimal evolution method in the MEGA 4.0 program. The tree was drawn to scale, and the percentage representations obtained from the bootstrap analysis were shown next to the appropriate branch point. Notice the clear separation of the *PAG *gene family into two groups, the modern and the ancient PAGs. Also notice the relatively shorter branch lengths in the modern *PAG *clusters compared to the ancient PAGs.

The phylogenetic relationships of various annotated *PAG*s in cattle were based on their predicted amino-acid sequences (Figure 1). The *boPAGs *were grouped into two distinct sub-classes, one of the two groupings, the 'modern PAGs' comprised the bulk of the *PAGs *represented in the build. They were found to be relatively tightly grouped with short branch lengths, consistent with the relative recent expansion of this cluster [29,32]. The others comprised a much smaller grouping (the ancient PAGs) and had relatively longer branch lengths and were loosely clustered.

Interestingly, the differences in phylogenetic relationships associated with this gene family correspond to differences in the pattern of the distribution of their message within the bovine placenta. Those members that are expressed by all trophoblasts are relatively ancient – having arisen more than 80 million years ago (MYA) [29]. In contrast, those *PAGs *whose expression is restricted to BNCs are relatively recently evolved genes that are predicted to have arisen 50–55 MYA [29]. This time frame corresponds approximately to the period in which the ruminant ungulates, with their unique synepitheliochorial type of placentation, are believed to have diverged from the swine lineage [49]. The BNCs are the hallmark of the synepitheliochorial placenta. These large cells, which comprise ~20% of the total trophoblast population, can fuse with uterine epithelial cells to form either a syncytium or short-lived trinucleated cells – depending on the species [50-54]. This fusion event is the extent of invasiveness in ruminant ungulates and is quite unique among eutherians [55-57]. This type of placentation probably developed from the completely noninvasive epitheliochorial placenta observed in non ruminants, such as camels and pigs [58,59]. Similarly, the origin of the *Artiodactyla *order itself has been estimated at about 83 MYA [49], a value that is very close to the estimate of when the *PAG *genes as a whole first began to duplicate. It is tempting to speculate that the burst of duplications that created to the *PAG *gene family initially were associated with the formation of the *Artiodactyla *order and they arose to fulfill a role distinctly required of the epitheliochorial placentae employed by these species. Likewise, the formation of the Modern PAG group may have been linked to the emergence of the sub-specialized synepitheliochorial placental type of the *Ruminantia*.

### Identification of repetitive and transposable elements within the *boPAG *genes

**Figure 2:**
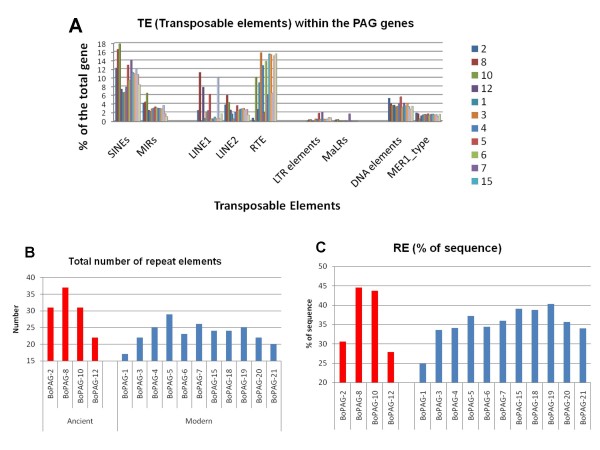
**Relative distribution of non-LTR (SINE, LINE), LTR and transposable elements (TEs) within several bovine *PAG *genes**. **A**. Each colored bar represents the relative distribution of the corresponding element in each *boPAG *gene. The TE elements were shown on the -X axis. The relative % of the sequence contributed by each element is shown on the Y-axis. The definition of the acronyms used in the figure is as follow: LTR: long terminal repeat; SINE: short interspersed element; LINE: long interspersed element; MIR: mammalian wild- interspersed repeat (sub-class of SINE); RTE: retrotransposable elements; MaLR: mammalian apparent long terminal repeat; MER: medium reiterated element. **B. and C**. show the cumulative total of the number of TEs, as well as the % contribution to the sequence of individual *boPAGs*. B: represents the cumulative total of the all the different kinds of the elements in individual *PAG *genes. **C**: shows the % make-up of the *PAG *genes by the TEs.

The incorporation of TE within genes can produce changes in the gene structure. Furthermore, the presence of TE in genes can provide insight into the evolutionary history of gene families. In order to evaluate the implications of transposition events on the *boPAG *genes, a preliminary evaluation was performed on the sequence of each *PAG *(including 3000 bp 5' and 3' of the coding regions of the gene). The distribution of TE in representative candidate *boPAGs *is shown in Figure 2A. The Repeat Masker software revealed that TEs were distributed only within the intronic and non-coding regions of the *PAG *genes. Consequently, the TEs are not directly influencing the reading frame of *boPAGs*.

The ancient *boPAG *group in general, and *boPAG-8 *and -*10 *in particular, had slightly more TE insertions than modern boPAGs (Figure 2B). The ancient *boPAGs *on average had 30.25 repeats incorporated into their sequences compared to 23.3 for modern *boPAGs*. *BoPAG-8 *had more elements incorporated into its gene than any other *boPAGs *analyzed with a total of 37.

This was closely followed by *boPAG-2 *and -*10 *with 31 repeats each (Figure 2B). Among the modern *boPAGs*, *boPAG-5 *had the largest number (29) of TE insertions, followed by *boPAG-7 *with 26 elements and -*4 *and -*19 *with 25 incorporated elements (Figure 2B). Regardless of the number of repeats incorporated, the total amount of sequence contributed by the introduced TEs did not noticeably differ between the two groups. For example, in the ancient *boPAGs *the average contribution to the actual size of the gene that was contributed by the elements was around 36.6% when compared to 35.2% in the modern *boPAGs*. Again, of all the *boPAGs*, the percentage of total gene length contributed by TEs was highest in *boPAG-8 *(44.5%) followed by *boPAG-10 *(43.6%) (Figure 2C). Interestingly, the average contribution of TEs to the size of the *boPAG *genes was much often less than the average of 45% in bovine genes in general, although the ancient *PAG *members, *boPAG-8 *and *boPAG-10 *had reached this proportion (Figure 2C) [46]. While the TEs have not influenced the ORF of *boPAG *genes, they may have a role in influencing the relative level of transcription of the genes or in shaping the evolution of the gene family. This investigation represents a necessary first step in understanding the role of these incorporated elements, a detailed examination is warranted to address their function in *PAG *gene family, which is beyond the scope of this report.

### Analysis of the promoters of *boPAG *genes

#### Selective pressure operating on *boPAG *promoter sequences

It was noted that the regulatory regions of the *boPAGs *do not share any conserved sequences with other genes whose expression is restricted to trophoblast (data not shown). This analysis sought to improve the understanding of the proximal promoters of *boPAGs *and identify any conserved elements within the family members. In order to better understand the selective pressures operating on the promoters, the observed p-distance of the promoters were plotted against the rate at which synonymous substitutions are occurring (dS) within the nucleotide sequences of each corresponding ORF. There were two principal assumptions within this analysis; these were that (1) dS of the exons of each analyzed gene pair was under neutral selection and would reflect the normal mutation rate for this chromosomal location, and (2) if the calculated p-distance within the promoter is equal to the dS of the exons, then the promoter is mutating at a rate that is expected for this location. If the observed ratio is above one, it was considered positive selection for nucleotide substitutions and if below one, it was purifying selection.

**Figure 3:**
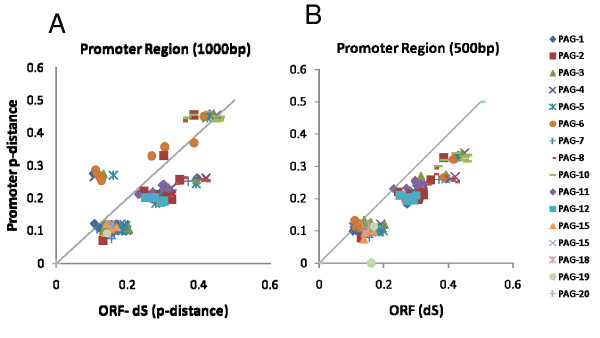
**The ratio of p-distance (p-dist) of the promoter regions versus predicted nucleotide mutation rate [calculated as dS (proportion of synonymous substitutions per synonymous site in the exons)] in pairwise comparisons for each *PAG *gene represented in the genome build**. A. Comparisons with the proximal 1000 bp of the promoter region. B. Comparisons with the proximal 500 bp of the promoter region. The p-distance of the promoters was shown on the Y-axis and the dS of their protein coding regions were displayed on the X-axis. The unique marks of a particular color and shape in the figure represent the pairwise comparisons of *boPAG *against each of the other *PAGs *included in the analysis. The listing of *PAG *genes and their indicators are shown in the legend.

The analysis was performed with two variable lengths of promoter sequence. When the p-distance v. the ORF for the proximal 1000 bp was mapped, all of the *boPAGs *were undergoing neutral to purifying selection (Figure 3A and 3B), with the exception of *boPAG-10 *and -*6*, which had ratios of more than one (Figure 3A). These promoters seemed to have accumulated more mutations than would have been predicted by molecular clocks. The analysis, when confined to the first 500 bp, generated similar results except that both *boPAG-6 *and -*10 *showed a ratio close to neutrality (Figure 3B). Overall, the *boPAG *promoters are being conserved, particularly in the first 500 bp upstream of the TSS (Figure 3B) implying that critical regulatory elements responsible for trophoblast expression may be positioned within this region.

### Multiple sequence alignment of the *boPAG *promoters for TE and conserved TF binding sites

#### Scrutiny of proximal promoter region for putative TEs

**Figure 4:**
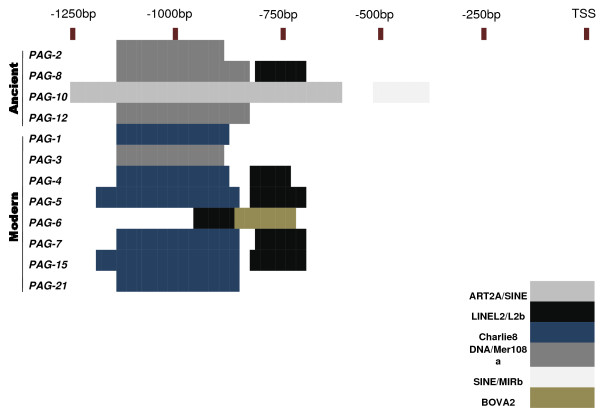
**Schematic representing TE insertion sites within the proximal 1000 bp of the promoters of *boPAG *genes**. Regions representing TE insertion sites within the multiple sequence alignment of the proximal promoter sequence of representative *PAGs *have been mapped into grid as per scale. Each colored block represents a corresponding TE insertion site within the respective region of a gene. The color codes representing the TEs are shown in the figure legend. Note that within the proximal 500 bp there are no insertional events within all the PAG genes except for *boPAG-10*. Within the grid TSS was noted as +1. Note that -1250 bp reflects -1000 bp. The discrepancy is due to gaps introduced during alignment of sequences.

To account for observed differences within the proximal promoter elements, the 1000 bp upstream of each TSS was assessed for the presence of repeat element insertions. The sequences of the promoters were aligned and the position and types of TE insertions were identified and mapped (Figure 4). Among all the *boPAG *promoters analyzed there were no TE insertions within the proximal 600 bp region with the exception of *boPAG-10 *which had a SINE (MIRb) insertion at -317 bp corresponding to -390 bp in multiple sequence alignment (TSS being base pair position +1) (Figure 4). An interesting observation was that the type of TEs detected in distinct *boPAG *promoters differed between modern and ancient *boPAGs*. In *boPAG-10 *(an ancient PAG) for instance, there was a long SINE-element insertion from -524 to -1066 bp (-631 to > -1250 bp in alignment) (Figure 4). The corresponding region was occupied by DNA element Charlie-8 in all modern *boPAGs *and an additional LINE element (L2) in *boPAG-4*, -*5*, -*7*, and -*15 *(Figure 4). In the ancient *boPAGs *there was a ~200 bp DNA MER-108 element upstream of -750 bp that was conserved in all the ancient *boPAGs*, with the exception of *boPAG-10*. Therefore, the two groups of the *boPAG *promoters deviated in the types of TEs that were inserted in their upstream regulatory regions, which also accounts for the large deviations in p-distances between the modern and ancient *boPAG *promoters (Figure 3A). Similarly, a lengthy SINE insertion was identified in the *boPAG-10 *promoter that was not found in any other *boPAG *promoters. The *boPAG-10 *promoter diverged considerably from the remainder of the *boPAG *promoters. The functional significance of these inserted TEs is not known, but a potential role for these elements in influencing the expression of *boPAGs *could not be ruled out.

#### Conservation of regulatory regions within the *boPAG *promoters

Based on previous reports, the *boPAGs *are known to exhibit differences in both their spatial and temporal expression patterns [13-15,28]. The availability of the full-length promoter sequences provided an opportunity to study putative regulatory elements that could potentially explain the observed differences in the temporal and spatial expression patterns.

**Figure 5:**
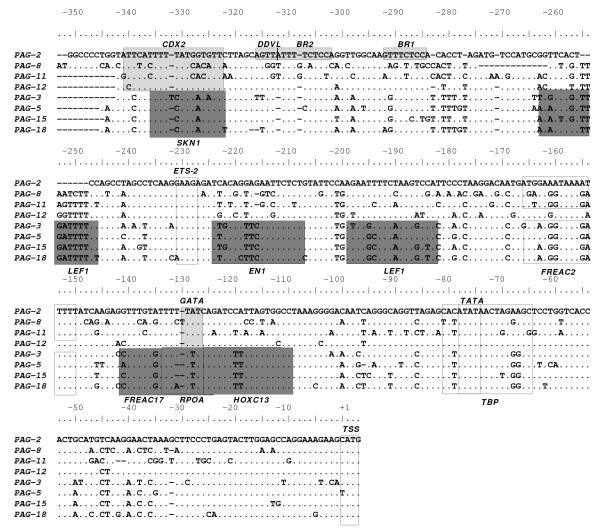
**Multiple sequence alignment of the proximal 350 bp of the promoter region showing conserved pockets bearing consensus recognition sequences for transcription factors (TFs)**. Regions conserved in at least 50% of the input sequence (4/8) that had putative TF binding sites were shown as boxed regions in the figure. Regions that are conserved across ancient *boPAGs *(*PAG -2,-8,-11 *and -*12*) were shaded in light grey and regions conserved across modern PAGs (*PAG-3,-5,-15*, and -*18*) were shaded in dark grey. Putative TFs capable of binding to the boxed residues were shown in the bottom of the alignment. The regions that are conserved across all the *PAGs *and the regions that are of importance for discussion were boxed (not shaded).

For this analysis, the first 1000 bp upstream of the TSS of various *boPAGs *was examined by using the DiAlign TF program of Genomatix-GEMS launcher. Among the aligned *boPAG*-promoter sequences, there were regions that were conserved in both the ancient and modern *PAGs *and, therefore, may contribute to trophoblast-specific expression. However, there were also a number of isolated conserved regions corresponding to consensus sequences for TF binding that were specific for ancient or modern *boPAGs *suggesting that the divergence of such elements could be responsible for the observed differences in the spatial distribution of the two *boPAG *groups. Examples of such regions within the first 350 bp of the TSS were boxed and listed in the Figure 5. Based on this analysis, conserved putative TF binding sites are highly prevalent in modern *boPAGs*. For example, there are predicted binding sites for these TFs: HOXC13 at position -109 to -125, RPOA (DTYPEPA) at -111 to -132, a FREAC17 at -124 to -141, FREAC2 at -149 to -166, LEF1 at -182 to -199 and -246 to -262, EN1 at -207 to -224 and SKN1 at -322 (TSS is +1). In addition, an atypical ETS site was conserved in all *boPAGs *and is located at position -227 bp to -230. Besides these sites, there were two tandem repeats (TTTCTCCA) 11 bp apart at positions -284 and -302 bp, respectively. Of these two repeats, the distal repeat was predicted to be recognized by DDVL (drosophila dorsal ventral factor) a homolog of vertebrate c-Rel TF. These repeats were conserved in most of the *boPAGs *and were referred to as 'bovine repeats' (BR); the presence of these repeats has been reported previously [60].

#### The relative distribution of *boPAG *ESTs in the bovine EST database

**Figure 6:**
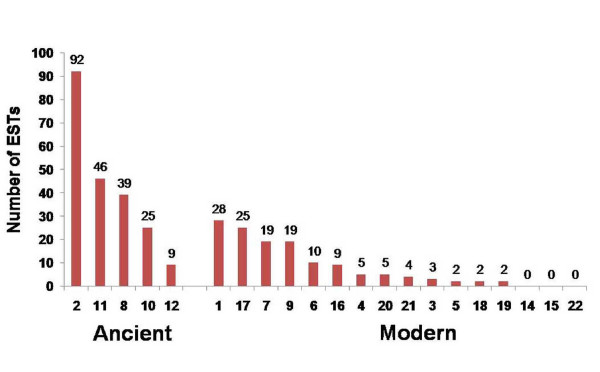
**The relative distribution of ESTs of individual *boPAGs *represented in the bovine EST database**. The total numbers of ESTs that matched the coding sequences with more than 98% nucleotide identity in at least 350 bp of query sequence were considered a match and were shown in the figure. Note the relative abundance of the ESTs corresponding to *boPAG-2 *in comparison to the other members of the *PAG *family.

In order to verify if apparent differences observed in the promoter sequence might be associated with the relative levels of transcription of various genes, the bovine EST database was searched to define the relative distribution of various *boPAG *transcripts. Of all the *boPAGs *that were investigated, *boPAG-2 *had the highest occurrence, with 92 ESTs represented in the database (Figure 6). The next most abundant member was *boPAG-11 *with 46 ESTs (Figure 6). Of the modern *boPAGs *that were assessed, *boPAG-1 *had the highest number of EST matches with 28, followed by *boPAG-17 *with 25 matches (Figure 6).

#### Real-Time PCR of bovine ancient PAGs (*boPAG -2, -8, -10, -11 *and -*12*)

**Figure 7:**
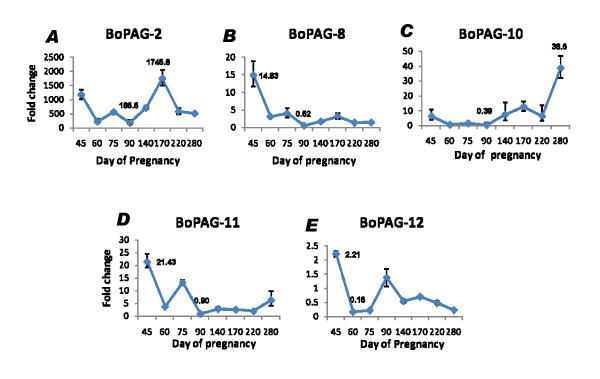
**Quantitative real time PCR results for ancient *boPAGs *(*boPAG -2, -8, -10, -11 *and -*12*)**. The relative fold changes compared to an external control gene (*YWHAG*) are shown. The different stages of pregnancy investigated are on the X-axis and the fold change on the Y-axis. Note the difference in scale between each panel showing the difference in message abundance between PAG genes.

As described above, *boPAG-2 *was an extremely abundant transcript. Therefore, follow-up experiments were performed to study the relative expression of *boPAG-2 *in comparison to its closest relative, *boPAG-12*, and to the other ancient bovine PAGs. Real time quantitative PCR of *boPAG -2*, -*8*, -*10*, -*11*, and -*12 *were performed and message abundance was assessed relative to an endogenous control transcript, *tyrosine 3-monooxygenase/tryptophan 5-monooxygenase activation protein*, *gamma polypeptide*(*YWHAG*). The source of RNA was obtained from placental cotyledons harvested at different stages of pregnancy, between d 45 and term. The relative amount of message for each target gene was graphed (Figure 7). *BoPAG-2 *was the highly abundant transcript relative to other ancient PAGs, while its most closely related family member, *boPAG-12*, was the least abundant under identical reaction conditions (Figure 7). Relative transcript abundance of *boPAG-2 *ranged from 186–1745 times greater than the control transcript, *YWHAG*, depending on the stage of pregnancy. In contrast, *boPAG-12 *message was much closer to that of *YWHAG*; its relative abundance varied from 0.16 to 2.21 that of the *YWHAG *transcript. The relative transcript abundance of *boPAG-8 *ranged from 0.5 to 14.83, *boPAG-10 *from 0.4 – 38.6 and *boPAG-11 *ranged from 0.9 to 21.4 times *YWHAG*-expression. Regardless of the stage of pregnancy that was examined, the transcript abundance of *boPAG-2 *was at least a 100 times greater than *boPAG -12 *and, when compared to other ancient PAGs, *boPAG-2 *message was at least 5 times greater (Figure 7). Finally, the relative profiles of each PAG transcript were distinct and they did not parallel one another. One interesting observation in particular, was that the relative temporal expression profiles of *boPAG-8 *and -*10 *were essentially opposite to one another. While the relative abundance of *boPAG-8 *was higher on d45 and was relatively stable across all other stages of pregnancy, *boPAG-10 *on the contrary had relatively low level of expression on d45 and had its highest level of expression at term.

#### Electrophoretic mobility shift assays

**Figure 8:**
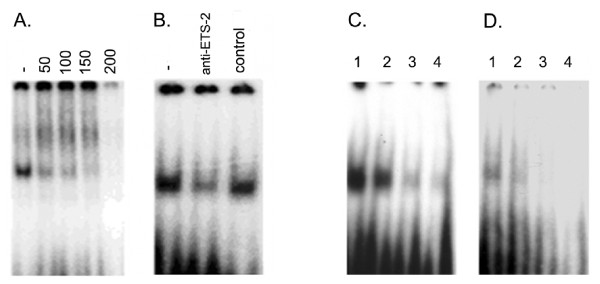
**Electrophoretic mobility shift assays demonstrating that the putative ETS site and the repeated elements in the *boPAG-2 *promoter are capable of binding proteins in trophoblast nuclear extracts**. A. Competition of ETS-2 binding activity (20 μg protein) with cold ETS-2 probe. Nuclear extracts were incubated with 1 μL of 50 pmol probe, in the absence or presence of the indicated molar excess of cold probe (indicated along the top). B. The ETS-2 complex composition was examined by depleting ETS-2 with an antibody specific to ETS-2. Preincubation of the ETS antibody with the nuclear extracts followed by binding reaction with the probe resulted in specific dissociation of the complex. Control: normal rabbit serum. C and D. Competition assays indicating specificity of association of, as yet unknown, TFs capable of binding to the unique bovine tandem repeats, BR1(C) and BR2 (D). Lane 1: labeled probe and nuclear extract; Lane 2: same as lane 1 except for addition of a 50-fold molar excess of unlabeled double-stranded oligonucleotide; Lane 3: 250-fold molar excess of unlabeled probe; Lane 4: 500-fold molar excess.

Since *boPAG-2 *was the most abundant transcript observed in the bovine genome, we set out to study its promoter in some detail. ETS-2 is a key TF involved in the regulation of numerous placenta-specific genes, such as interferon-tau (*IFNT*) [61] and the human chorionic gonadotropin (*hCG*) beta subunit [62]. As mentioned previously, an ETS-2 site is present in all *boPAG *promoters (Figure 5), including *boPAG-2*, and may be critical to its transcriptional regulation. Competition and super shift assays (Figure 8A, and 8B) were performed with ^32^P-labeled oligonucleotides representing the putative ETS site from -226 to -229 (Figure 5). We utilized nuclear extracts from JAr human choriocarcinoma cells for this experiment, since nuclear extracts from bovine placental samples couldn't be obtained. EMSA's with nuclear extracts from JAr cells, which constitutively express ETS-2, indicated the presence of a protein(s) capable of specific association with the oligonucleotide probe. The complex could be competed away by excess unlabeled probe and could be decreased by the addition of an anti-ETS antibody. Likewise, the unique bovine tandem repeats (BR-1 and -2) which were reported previously and were found to be conserved across most of the PAGs [60] were also investigated by EMSAs to determine if proteins present in human JAr cells are capable of binding to these repeats. A specific complex was identified that could be competed away with an excess of non radiolabeled specific competitor (Figure 8C and 8D) implying that these repeats could possibly bind to endogenous TFs in placenta. Although, the experiments were conducted with cells of chorionic or placental origins from human, we anticipate that the observed results would also hold true with bovine placental samples.

## Conclusion

In conclusion, the bovine genome sequencing project has facilitated an increased understanding of the *PAG *promoters and *PAG *gene organization. The *boPAG *gene family was verified to be rather large and complex with 18 functional and 14 probable pseudogenes (no ESTs have been found for these). The analysis of the proximal promoter regions encompassing 500 bp upstream of the TSS in all these genes revealed a high level of conservation between these genes suggesting that crucial transcriptional regulatory elements likely reside in this region. *In silico *analysis revealed that while there were regions of conservation shared by all *PAGs *(probably influencing trophoblast specific expression of these genes), there were also elements that were present only in the modern or the ancient PAGs. Indeed, most of these class-specific elements tended to be observed in the modern *PAGs*. Since these regions contain recognition sequences for putative TFs; it is attractive to speculate that these putative regulatory sequences could contribute to the observed differences in spatial and temporal expression between PAGs. We have shown by bioinformatics and experimental analyses that *boPAG-2 *is the most abundant of all the *PAGs *and that the unique ETS-2 and DDVL group of TFs were potentially involved in the regulation of this gene. While the role of these particular TFs was implicated by the EMSAs, the putative role of LEF1- a mediator of Wnt signaling, whose consensus recognition sequence is shared in all modern PAGs is also an interesting candidate for future investigation [63,64]. Likewise, CDX2, which has a demonstrated role in trophoblast lineage specification and regulation of trophoblast expressed genes, has a conserved putative binding site among all ancient PAGs and is another such likely candidate for *PAG *transcriptional regulation [65,66]. An analysis of the actions of ETS, and other TFs, in regulating the different spatial and temporal expression patterns of the *PAG *genes will likely be an interesting and fruitful endeavor. Finally, with the ongoing efforts to modify and further refine the genome build, we are positioned to further our understanding of the organization and evolution of the *PAG *gene family.

## Abbreviations

PAG: Pregnancy-associated glycoprotein; *boPAG*: bovine pregnancy-associated glycoprotein; AP: aspartic peptidase; TSS: Translational start site; TF: transcription factor; TE: transposable elements; ORF: open reading frame.

## Authors' contributions

BT and JG conceived and designed the project, performed bioinformatics, and prepared the manuscript. BT performed the Real-time PCR experiments. AMW assisted in repeat masker analysis of PAG genes and analysis of selection pressures on promoters and revision of manuscript. All authors read and approved the final version of the manuscript.
